# Road to Paris 2024: force-velocity profile in different speed climbers’ abilities

**DOI:** 10.5114/biolsport.2024.131824

**Published:** 2023-10-16

**Authors:** Violeta Muñoz de la Cruz, Víctor Rodrigo Carranza, José María González Ravé

**Affiliations:** 1 Sports Training Lab: Sports Performance Research Group (GIRD) University of Castilla-La Mancha, Toledo, Spain

**Keywords:** Sport climbing, Physical performance, Squat, Pull-up, Muscle power

## Abstract

Speed climbing will be a new discipline in Paris 2024. The physical requirements of speed climbing are different from the other climbing modalities due to the short event time requiring higher level of strength and power. These parameters have been measured through the Force-Velocity (F-V) profile in different climbing disciplines. However, there are no known results evaluating different speed climbing abilities to establish whether F-V relationship is a determining factor between performance levels. The purpose of this study was to evaluate the upper and lower limbs F-V profile in different speed climbing abilities considering sex. Twenty-six speed climbers were divided into two groups based on their level of performance: international level (men n = 7 and women n = 2) and national level (men n = 8 and women n = 7). Participants performed pull-ups and squat incremental tests and F-V profile variables [Maximum theorical values of force (F0), velocity (V0) and power (P_max_)], one-repetition maximum value (1RM) and %1RM where peak power was expressed were collected using a linear encoder. There were significant differences in F_0_, relative force, %1RM where peak power was expressed, and 1RM in pull-ups (p < 0.05) between groups. However, there were not significant differences between groups in squat variables. No significant sex differences were found in any variable. There were moderate-strong correlations between running time and 1RM (pull-ups and squat), F_0_ and FV-slope (pull-ups) (p < 0.05) analyzed in the whole group. In conclusion, F_0_ and 1RM in pull-ups were significantly higher in international climbers. Therefore, national climbers should focus their training on improving force by training with heavy loads. Additionally, squat F-V profile variables do not seem to be as important as in the pull-up for performance.

## INTRODUCTION

The 2024 Paris Olympics will once again feature sport climbing. In the previous event (Tokyo, 2020), the three disciplines (lead, bouldering and speed climbing) were included as one combined competition, not allowing the specialization of the climbers in a specific discipline [1]. This time, the Olympic program will separate sport climbing into two disciplines, speed climbing and the combined (lead and boulder). In fact, speed climbing has gained significant attention [1] as many climbers have started to specialize in this discipline in preparation for the Paris 2024 games. This discipline consists of climbing a standardized wall of 15.5 meters high and 5º inclination with 20 hand holds and 11-foot holds in the shortest time possible after a start signal of 3 tones separated by one second [2].

Speed climbers require higher levels of strength, anaerobic power, and speed compared to climbers of other disciplines [3–5]. Traditionally, climbing training programs have primarily focused on developing upper limb capabilities [6]. However, recent studies have highlighted the significant influence of the lower body in speed climbing, as it demands different abilities compared to other climbing disciplines, founding strong correlations between running time and leg power output and hypertrophy [7–9].

A key factor in power-based sports is the Force-Velocity (F-V) relationship [10]. This linear relationship is explained as the ability to produce force at different velocities of motion that are inversely related to each other [11]. For instance, two speed climbers can achieve the same power with two distinct F-V profiles, differing in the amount of force or the amount of velocity. Thus, the F-V profile can be used by coaches to orientate the strength training according to the estimated value of maximum force (F_0_), the estimated value of maximum velocity (V_0_) and the resulting estimated value of maximum power (P_max_) [10].

The F-V profile can vary based on the physical requirements of the sport [12]. To the best of our knowledge, there is only one study analyzing the pull-up F-V profile in speed climbers [13]. Levernier et al. [13] compared climbers from the three disciplines obtaining significant differences in several F-V profile variables. However, this information is no longer relevant as sport climbing will not be a combined event anymore. Additionally, there is a lack of research comparing the squat F-V profile characteristics across different climbing performance levels and between sexes. Therefore, the aim of the current study was to determine the F-V profile in speed climbers in the upper (pull-up) and lower limbs (squat) in different climbing abilities (international and national level) considering sex. We hypothesize that there will be differences in the F-V profile depending on the performance level of the climbers and depending on the sex with international men showing better results than women and national climbers.[14]

## MATERIALS AND METHODS

### Participants

A total of 26 speed climbers (women n = 11 and men n = 15) from different countries (Spain, Italy, Austria and Ecuador) participated in this study. They were divided into two groups based on the classification criteria established by Mckay et al. [14]: International level (n = 9;7 men and 2 women) where 3 climbers obtained a medal in an international event at least once in their life and national level (n = 17; 8 men and 7 women). Detailed information about the participants can be found in Table 1. None of them had been injured in the last year and they signed an informed consent form before the test which explained the procedure and the risks involved. The study was performed in accordance with the principles of the Declaration of Helsinki (October 2008, Seoul), and the experimental protocols were approved by the local ethics committee (CEIC).

**TABLE 1:** Participants characteristics.

Level	n	Age (years)	Body weight (Kg)	Best time (s)
Men international	7	17.7 ± 7.41	67.1 ± 3.57	6.72 ± 0.72
Women international	2	26.0 ± 0.00	61.1 ± 3.82	8.11 ± 0.71
Men national	8	16.0 ± 2.00	63.9 ± 7.74	8.83 ± 1.15
Women national	9	18.9 ± 7.41	63.1 ± 9.49	11.70 ± 1.69

International group: Climbers who have participated in international championships. National group: Climbers who perform at national competitions in their country.

### Instruments

Incremental tests in both exercises were performed using a validated linear encoder (ADR encoder, Toledo, Spain) [15, 16]. This device uses linear motion translation technology to measure the mean propulsive velocity, which is defined as the mean velocity from the beginning of the concentric phase until the acceleration of the bar is less than gravity [17]. The encoder is fitted with a cable that is attached to the bar (in the case of squats) or to the back part of the harness (in the case of pull-ups) which makes possible to measure the linear displacement during the exercise.

### Procedures

This study is a comparative descriptive design in which the F-V profile of international and national level speed climbers was analyzed and compared. All participants were instructed not to consume caffeine in the previous 12 hours before the test [18]. The menstrual cycle of the female climbers was not considered. However, recently it has been shown that force, velocity and power output remain very similar in all menstrual cycle phases [19] and particularly in the squat exercise [20]. The protocol was adapted from Muñoz-López et al. [21] although not exactly the same one as we analyzed loads under 70%1RM, and we did not get to the 1RM. Participants performed a standardized warm-up of 15 minutes which consisted of preparatory exercises (i.e., scapular retractions and glenohumeral joint external rotations) and one set of 5 unweighted pull ups (instructed to go “as fast as possible”) before the first test. The first test carried out was incremental pull ups test in which participants started with a load of 0 kg extra-weight. Afterward, they increased the load by 5 kg or 10 kg (based on the drop of velocity from the previous load) until they reached an execution velocity of approximately 0.35 m · s^−1^. They performed two repetitions per load, where the one with a higher mean propulsive velocity was recorded. Participants started to do only one repetition when the mean propulsive velocity was under 0.5 m · s^−1^. The recovery time between each load attend was 3 minutes [22]. Concerning the body position, the climbers performed pull-ups placing their hands in pronation and at a distance of 1.5 their biacromial breadth (cm) following the posture protocol established by Levernier et al. [13]. The pull-up was considered valid if the chin went above the bar and if the participants did not intentionally use the legs to help themselves going up. Additionally, every repetition started with both arms fully extended and the exercise was performed linearly and controlling trunk movements in order to provide accurate distance measurements when using the linear encoder.

Between pull-up and squat tests, they were allowed to rest for 15 minutes. Before starting the squat incremental test, they were instructed to conduct again a standardized warm up of 10 minutes which consisted of hip mobility exercises and one set of squats at a velocity faster than 1 m · s^−1^ until 20% of velocity loss. The protocol for squat test was adapted from Bachero-Mena et al. [23]. The test started with a load of 20 kg and from this point on, they increased by 10 kg until they reached a velocity close to 0.45 m · s^−1^. They performed two repetitions with each load until they lifted the load to a velocity of less than 0.7 m · s^−1^, from here on they lifted each load one time. The recovery time was 3 minutes between trials [22]. The squat was back squat and parallel (when the femur is parallel to the ground), so we adjusted the structure’s supports to make the barbell touch the supports when the participant’s thigh was parallel to the ground. The climbers were instructed to descend in a controlled manner, touch the supports with the bar for approximately half a second and to lift the load as fast as possible. In the pull-up test, a range between 58.8 ± 7.8% – 81.7 ± 5.9% considering F_0_ was analyzed while in the squat exercise the range was from 53.6 ± 6.9% – 77.3 ± 6.3%.

In order to correlate pull-up and squat performances with the speed climbing ability we used an official running time (official wall and chronometer) performed in an official competition within seven days before or after pull-up and squat tests day.

### Data analyses

The F-V relationship was determined by least-squares regression analysis. Tests with a correlation lower than r^2^ = 0.95 between loads and velocities were discarded. Thus, four pull-up tests (One from international group and three from national group) and six squat tests were removed from the sample (One from international group and five from national group). We obtained F_0_ and V_0_ from the intercepts of the F-V curve regression with the axes X and Y. P_max_ was obtained through the following formula: (F_0_ · V_0_) / 4 [24], while peak power was the highest power expressed during the test. F_0_ and P_max_ were analyzed considering body weight. In order to estimate 1RM values, we considered the velocity proposed by Muñoz-López et al. [21] for pull-ups (0.26 m · s^−1^) and Sánchez-Medina et al. [25] for squat 1RM (0.32 m · s^−1^).

### Statistical analyses

All data are presented as mean ± standard deviation (SD). The normality was checked before any analyses using Shapiro-Wilk test and data followed a normal distribution (p > 0.05). A significant level of p < 0.05 was adopted. A Two-way ANOVA test was conducted to examine differences in each dependent variable considering sex and level of performance. Pearson correlation test was carried out to analyze if there was a correlation between any of the dependent variables with running time. The statistical tests were processed with the use of the software Jamovi 2.3.18.0 for Windows.

## RESULTS

No significant differences (p > 0.05) were found in any of the dependent variables between sexes. There were significant differences (p < 0.05) in the 1RM, relative force and F_0_ in pull-ups between the international and national groups. In the squat exercise, no differences were found in any of the independent variables (Table 2).

**TABLE 2:** ANOVA between performance levels in pull-up and squat exercises.

	LEVEL	1RM (Kg)	Relative Force (Kg · BW^−1^)	F_0_ (N · Kg^−1^)	V_0_ (m · s^−1^)	^P^max (W · Kg^−1^)	FV slope (N · (m · s)^−1^)	%1RM (Peak power)
Pull-ups	International	47.80 ± 13.1**	0.68 ± 0.12**	19.50 ± 2.44*	1.93 ± 0.29	9.35 ± 1.07	-690 ± 206	59.70 ± 4.22*
	National	27.00 ± 8.25**	0.43 ± 0.13**	16.20 ± 2.13*	2.07 ± 0.35	8.33 ± 1.20	-511 ± 137	70.60 ± 6.33*

Squat	International	111 ± 22.2	1.60 ± 0.25	29.50 ± 4.23	1.93 ± 0.36	14.00 ± 1.46	-1090 ± 356	44.40 ± 9.57
	National	89.80 ± 20.1	1.40 ± 0.22	27.40 ± 3.20	2.21 ± 0.47	14.80 ± 1.90	-837 ± 285	48.50 ± 10.70

Note. 1RM = One repetition maximum; Relative Force = 1RM kg / bodyweight kg; F_0_ = Maximal estimated force/ bodyweight Kg; V_0_ = Maximal estimated velocity; P_max_ = Maximum estimated power/ bodyweight Kg; FV slope = Force-velocity slope; %1RM (Peak power) = Percentage of 1RM in which peak power is expressed;

* p < 0.05;

** p < 0.001

**FIG. 1:**
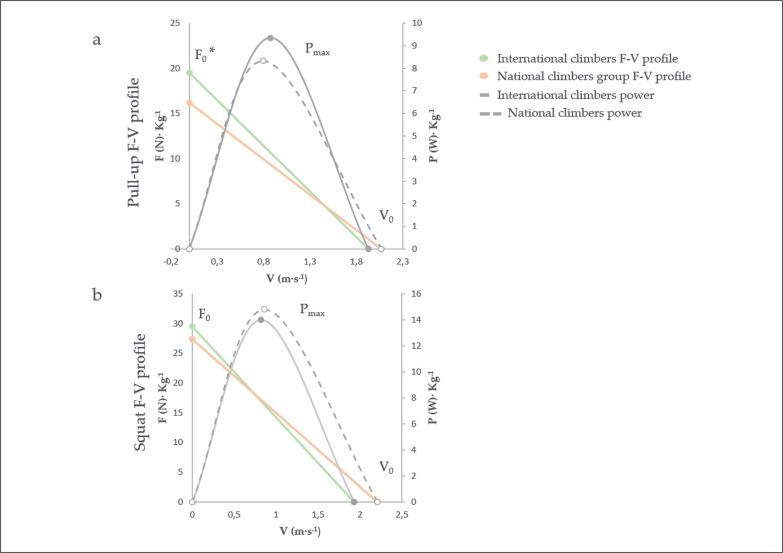
a. (Pull-up F-V profile) b. (Squat F-V profile) F · kg^−1^ = Force · bodyweight^−1^; V = Velocity; P · kg^−1^ = Power · bodyweight^−1^; F_0_ = Maximal estimated force · bodyweight^−1^; V_0_ = Maximal estimated velocity; P_max =_ Maximal estimated power · bodyweight^−1^; * p < 0.05.

These results have been graphically expressed in the figure 1.a and 1.b, showing the comparison between national and international level climbers regarding average values of F_0_, V_0_ and P_max_.

Analyzing the percentage of 1RM at which participants reached peak power, we found significant differences in the pull-up exercise, with international level athletes reaching it at 59.7 ± 4.22% of 1RM and national level athletes reaching it at 70.6 ± 6.33% of 1RM, although 88% of climbers achieved this with their own body weight. In the squat we found no significant difference between levels in the percentage of 1RM in which they express their peak power where international level climbers obtain it at 44.4 ± 9.57%1RM and national level climbers at 48.5 ± 10.7%1RM.

No sex differences were found in any variable except from squat P_max_ (relative to bodyweight). Male speed climbers obtained significantly higher values (p < 0.05) than female climbers.

In addition, we analyzed the correlation between the different variables obtained from both tests and the time (s) spent on the wall (running time) where we found significant and strong correlations between this time and 1RM (pull-up and squat), F_0_ (pull-up) and FV slope (pull-up) (Table 3).

**TABLE 3:** Significant Pearson correlations between running time and pull-up and squat variables.

Dependent variable	Correlation with running time
1RM pull-up	-0.74
1RM squat	-0.50
F_0_pull-up	-0.70
FV slope pull-up	0.61

Note. 1RM = One repetition maximum; F_0_ = Maximal theorical force; FV slope = Force-velocity slope.

## DISCUSSION

The aim of this study was to determine the F-V profile in speed climbers in upper (pull-up) and lower limbs (squat) in different climbing abilities (international and national level climbers) and sexes. In relation to our hypothesis, there were significant differences in F_0_, relative force, 1RM and percentage of 1RM where peak power is reached in pull-ups between groups. However, there were not significant differences between groups in squat F-V profile variables. In addition, no differences were observed between sexes in the pull-up and squat exercises.

Strength and power characteristics have been previously analyzed in national level speed climbers [5] and international-elite speed climbers [3, 7, 26]; however, the F-V profile has barely been investigated and differences between performance levels have not been analyzed yet in speed climbing. Moreover, previous studies examined a small sample size and were conducted only with elite climbers, leaving a gap of knowledge on the different characteristics of climbers who perform at national level and who are on their way to high performance. For this reason, in our study, climbers from different countries were tested to obtain data from a bigger group who performed at different levels and therefore a larger sample that would provide us with a broader view.

Upper body exercises including pull-up exercise have been widely studied in sport and rock climbing [27]. Speed climbers have usually practiced other climbing disciplines and perform it in their training. However, not knowing the F-V profile of an athlete implies difficulties in orienting training towards more speed work or more strength work within strength training. Levernier et al. [13] compared the pull-up F-V profile in elite climbers of the three disciplines and found significant differences between boulder and speed climbers in V_0_ and P_max_ (where boulder climbers had higher values) but did not find significant differences in F_0_. Therefore, the F-V profile of speed climbers was not less force oriented than in other disciplines. Anyhow, we cannot compare these results with our study as we did not analyze climbers from different disciplines.

Our study presented a significant difference between levels in pullup F-V profile, where F_0_ was higher in international level. This could mean that pull-up training in speed climbers, especially in national level, might be oriented to maximize the force component (training with heavy loads) instead of performing ballistics movements (velocity component) [28]. In addition, 1RM and relative force values were significantly higher in international climbers.

Relations between power, force and velocity in lower limbs have previously been studied, where the correlation between running time and velocity component has been found to be stronger than running time and force component [7]. Nevertheless, this correlation was made analyzing only one jump (countermovement jump (CMJ)) per participant and not through an incremental test with different loads (F-V profile). Regarding our results, no differences were found between performance levels in the F-V profile variables in the squat exercise.

Nonetheless, previous research has shown the importance of lower limbs in speed climbing [4, 5, 7]. Strong correlations have been confirmed between the height of the jump in countermovement jump or vertical jump and the level of performance in the climbing wall [4, 26, 29], although in some of these studies the correlations were not significant. In our research we found a moderate correlation (r ≥ 0.5; p < 0.05) between running time and squat 1RM, however correlations between pull-up variables and running time were greatly stronger (Table 3). Therefore, in the squat exercise it resembles that the most important variable is to have a high 1RM value regardless of whether it is through velocity or force component. The parallel squat exercise may not adequately display the climbers’ lower limb skills. It has been found that the dynamic movements during the climbing route present a pattern of movement and a force-time profile very similar to that of the squat jump [26]. Future studies could investigate the correlation between the F-V profile of squat jump exercise and the performance on the wall.

The range of 1RM percentage at which the peak power is expressed in different exercises has been studied by several authors [30, 31] proposing that training with this percentage can provide greater benefits to improve power performance. However, studies analyzing this percentage in the pull-up exercise are scarce. Muñoz-López et al. [21] found that the load with which they expressed the greatest power was 70% of the 1RM and this amount usually coincided with the first load of the test, which was their own body weight. However, they concluded that this load was not light enough to find peak power and that this would be found by using a resistance that allows the body weight to be reduced (i.e., assisted training using elastic bands or conditioning machines). In our study, we obtained similar results, since most of the climbers obtained their peak power with the first load (body weight), except for some of the participants with the best 1RM who obtained it with a load of 10 kg. Likewise, Muñoz-López et al. [21], national level climbers obtained peak power in a range close to 70%1RM, however, international level climbers expressed it in a significantly lower percentage close to 60%1RM. This could be because international level climbers had a higher 1RM than national level climbers and the participants in Muñoz-López et al. [21].

On the other hand, the squat exercise has been more studied. It has been found that elite athletes express their peak power in the squat exercise between 30–70% of the 1RM [30]. Our results showed that national and international level climbers expressed the highest power value in this range, although generally closer to 30% than 70% of 1RM. In fact, there is no significant difference between the percentages of 1RM at which they obtained peak power between performance levels.

Regarding to sex differences, distinctions [32, 33] and similarities [34] in F-V profile characteristics have been evaluated in different physical exercises. One of the factors that can influence the F-V profile sex discrepancies could be the body fat-free mass [35]. Although we took body weight into account in the calculation of the F-V profile variables, body composition data were not considered. This might be considered a research limitation and we suggest measuring body composition values in future studies of this topic to evaluate possible differences between sexes. However, there were significant sex differences in squat power despite no sex differences in force or velocity components. Similarly, previous studies have reported results in other speed related sports, obtaining greater values for men in peak power [36]. This can be explained as commonly, women have less muscle volume than men in this kind of sports and the among of muscle mass is the major determinant of joint torque [37].

In addition, other limitations were found in this study. Previous studies have shown that a doble-hyperbolic equation between force and velocity fits more properly than the common linear one. In fact, using a linear equation can provide invalid and underestimated data respecting V_0_ and P_max_ [11]. However, the practical application of the linear equation may reduce the limitations in specific contexts, principally when data collected is above 45% F_0_ as in the present study. Furthermore, multi-joint exercises, such as those carried out in our research, may present a decrease in the curvature magnitude compared to single-joint exercises [11].

This study showed the abilities differences in F-V profile in pullups and squats in speed climbers where pull-up F_0_ was found significantly higher in international speed climbers. The information provided could be used by coaches and sport scientists in order to improve speed climbers’ performance. In addition, further studies could consider our findings and keep on studying this new Olympic discipline which its scientific background is scarce.

## CONCLUSIONS

The pull-up F-V profile displayed different between performance levels due to a higher F_0_ value in international climbers. Therefore, the training of pull-ups in national level climbers could be considered to be focused on increasing the force component through heavy loads training. The squat F-V profile stayed similar at both performance levels although it is possible that this exercise does not adequately represents the lower body capabilities of speed climbers.

Forthcoming studies should analyze F-V in squat jump as it has been shown that this exercise expresses climbers’ skills more adequately [26]. In terms of peak power, national level climbers obtained it in a higher percentage of 1RM than the international level climbers in the pull-up exercise. Additionally, there were not significant sex differences in any F-V profile variables except from squat P_max_, however fat-free mass should be considered in future research. Nevertheless, the science in this new Olympic discipline remains very young and more scientific studies are needed to provide vital information for the sake of adequate and specific training programs.

## Data availability statement

The data that support the finding of this study are available from the corresponding author upon reasonable request.
